# Nursing students’ knowledge and skills on children’s environmental health in Ethiopia: A cross-sectional Study

**DOI:** 10.1371/journal.pone.0336813

**Published:** 2025-11-13

**Authors:** Werku Etafa, Wandimu Muche, Dereje Temesgen, Dawit Tesfaye

**Affiliations:** Department of Pediatrics and Neonatal Nursing, Institute of Health Sciences, Wollega University, Nekemte, Ethiopia; Mizan-Tepi University, ETHIOPIA

## Abstract

**Background:**

Children’s environmental health encompasses a wide range of factors that impact the well-being of children, including physical, chemical, biological, and social elements in their immediate environment. Safeguarding children from harmful substances is the crucial role of nursing students. Nursing students play a vital role as valuable resources for individuals, families, communities, and policymakers. This study aimed to assess the knowledge and skills of nursing students concerning children’s environmental health at academic institutions delivering nursing programs in Nekemte town, Ethiopia.

**Methods:**

An institutional-based cross-sectional study was conducted in Nekemte town from 1^st^ to 30^th^ August, 2023, among 634 randomly selected nursing students using standardized questionnaires: Children’s Environmental Health Knowledge and Skills Questionnaire (ChEHK-Q and ChEHS-Q). Data were entered into Epi Data 3.1 and analyzed in SPSS 25, with linear regression applied to identify predictors of knowledge and skills at 95%CI and p-value<0.05 in multivariable linear regression.

**Results:**

Most nursing students had insufficient and poor knowledge (79%) of children’s environmental health, and over a third (34%) showed insufficient skills. The study also found a reciprocal relationship: students’ skills (β = 0.03, CI: 1.01–1.04, p = 0.01) and age (β = 0.06, CI: 1.02–1.10, p = 0.001) predicted their knowledge, while knowledge (β = 0.06, CI: 1.01–1.11, p = 0.01) and age (β = 0.05, CI: 1.02–1.09, p = 0.002) predicted their skills.

**Conclusions:**

The study concludes that nursing students possess limited knowledge and skills in children’s environmental health. It is suggested to integrate children’s environmental health into nursing curricula, strengthening pediatric and neonatal education, and conduct further research to address the gap.

## Introduction

Environmental health is vital to any comprehensive public health system that focuses on the connections between people and the environment, promotes health and well-being, and helps create healthy, safe communities [1]. All things in the environment exist in solid, liquid, or gaseous form, interacting with pollution and human beings [2]. The discipline of environmental health works to reduce exposure to harmful substances in the air, water, soil, and food necessary to protect children [1,2] (Association, 2019 #288;Moeller, 2011 #294;Moeller, 2011 #294). It focuses on environmental hazards (environmental risks, water, hazardous wastes, climate change, hygiene and sanitation, toxic metals, and chemicals) that affect children in several ways [3].

Environmental toxicants can lead our children to profound adverse health outcomes, either from the parents or the environment [4]. In the sequence of descriptive age periods, children are sensitive and highly affected by environmental hazards due to parental exposure before conception, exposure in the uterus, or exposure after birth [5]. Biologically, although children are small, they eat more food, breathe more air and drink more water than adults per their weight [6]. Children are at high risk for environmental hazards mainly due to immaturity (developmentally and immunologically); putting their hands or objects [5]in their mouth and playing on the ground are the most common behaviors that increase their exposure to environmental hazards [7]. Environmental health risks are shared mainly at home, school, and community [8].

The Sustainable Development Goals (SDG) elements: good health and well-being, clean water and sanitation, and climate actions are interconnected and imperative triangles for reducing neonatal and childhood mortality [9]. Globally, the environment contributes to about 13.7 million (24%) deaths, including one in four deaths of children under five years of age and more than a quarter (23%) of disease burdens [10]. The World Health Organization (WHO) reported that 1.8 billion (93%) children under 15 breathe polluted air, putting their health and development at serious risk [11]. Blood lead poisoning (at or above 5micrrogram/deciliter, ≥ 5 µg/dL) was in about 800 million (about one in three) children mainly in low- and middle-income countries (LMICs) [12].

Children endure a disproportionate share of environmental hazards and their immediate and long-term effects, resulting in disease, impairments, and mortality [13]. Environmental risk factors (foodborne toxins, poor sanitation, dirty homes, poor quality cooking fuels, dirty and poor local waste disposal) contribute to stunting in children [14]. Environmental hazards lead to long-term consequences such as violent behaviors, poor physical growth, and difficulties in mental activities, including attention deficits and low academic performance [9].

Efforts to clean up the environment could reduce childhood mortality by 25%. Each year, nearly two million deaths among children under five are linked to environmental factors [15]. Nurses constitute about 59% of the health professionals in the world and cover the significant portions of healthcare delivered to individuals [16]. It is the responsibility of nurses to safeguard the community from the health consequences of environmental hazards [17].

All nurses should be knowledgeable about the fundamental pathways of exposure to environmental hazards, prevention and control strategies, as well as effective interdisciplinary interventions [18]. Nursing education is required to include information about climate change at all levels to equip present and future nurses with the knowledge needed to provide care [19]. Nurses are essential for achieving Sustainable Development Goals 3 (SDGs 3), which encompass various critical health objectives. They play a pivotal role in ensuring universal health coverage, addressing mental health issues, and managing non-communicable diseases. Additionally, nurses are vital in ensuring emergency preparedness and response, promoting patient safety, and delivering integrated, people-centered care. Their work embodies the commitment to “leave no one behind,” ensuring that all individuals receive the care and support they need to lead healthy lives [16,20].

The Lancet Countdown on Climate Change and the International Council of Nurses have emphasized the importance of integrating environmental health into nursing education, practice, and policy [21]. This integration is vital for equipping nursing professionals with the essential knowledge, skills, and insights necessary to navigate clinical practice in the face of climate change [19]. Innovative strategies to incorporate Education for Sustainable Development principles into nursing curricula have shown positive effects on attitudes towards climate change and sustainability, resulting in significant improvements in knowledge [22]. Consequently, nursing students are expected to enhance their understanding and skills to prevent and address the health impacts of environmental hazards on children’s health [23].

In Ethiopia, there exists a significant gap in the nursing curriculum regarding climate and environmental health (CEH), as noted by nurse researchers and educators. Despite the escalating negative impacts of environmental health issues over time, there remains a minimal emphasis on incorporating CEH into the nursing curriculum. This lack of integration hinders the ability of nursing professionals to adequately address the challenges posed by environmental health in their practice.

Nursing students represent a crucial resource for individuals, families, communities, and policymakers, particularly in the development of curricula. In Ethiopia, a significant portion of the population, along with their children, resides in rural areas where primary healthcare services are predominantly provided by nurses, who are the main healthcare professionals in these regions. However, based on researchers’ experiences, there is a notable absence of content related to children’s environmental health in nursing education, and training in this area is virtually non-existent in the country. Additionally, there appears to be no studies aimed at evaluating the knowledge and skills of nursing students concerning children’s environmental health. This study aims to serve as a baseline for nursing educators, researchers, and policymakers in Ethiopia, focusing on assessing the knowledge and skills of future nurses regarding children’s environmental health.

## Methods

### Study area, design and period

The research was conducted at Wollega University (WU), Rift Valley University (RVU), and New Generation University College (NGUC), located in Nekemte town, Ethiopia, all of which offer degree programs in the field of nursing. An institutional-based cross-sectional study design was implemented from the first August to 30^th^ August, 2023, involving nursing students enrolled in degree programs at the mentioned institutions. Approximately 2780 nursing students were enrolled in the academy.

### Source and study population

The source population comprised all nursing students enrolled in universities and colleges located in Nekemte town. The study population consisted of nursing students from the source population who met the inclusion criteria.

### Eligibility

The inclusion criteria consist of nursing students who are enrolled in a recognized nursing program, have completed at least one hospital-based practical course, and have successfully finished the prerequisite coursework, including the fundamentals of nursing, relevant to the skills or knowledge being assessed.

### Sample size and sampling procedure

To determine the sample size, the single population proportion formula was used, considering a 50% proportion (p), a 95% confidence level, and a 5% margin of error. The calculated sample size was 423, and after accounting for a 10% non-response rate and a design effect of 1.5, the final sample size was 634. A sample of 634 nursing students was randomly selected from three academic institutions (WU, RVU, and NGUC) after proportional allocation to each institution based on their study year. The students were identified from their respective registrar offices and selected based on inclusion criteria using systematic random sampling.

After estimating the required sample size proportionally, we identified the number of students and sections based on information obtained from each academic center’s registrar office. Subsequently, the sections needed for the study were selected using a lottery method. This process continued until the estimated sample size from each section was fulfilled, with participants being selected through the same lottery method.

### Variables

The dependent variables were the knowledge and skill of the students in Children Environmental Health (CEH). Independent variables included demographic and educational factors such as age, gender, year of study, study program, and received training (lecture) about CEH.

### Operational definitions

- Knowledge: The knowledge questions were utilized to categorize the level of knowledge as excellent, very good, good, insufficient, or poor based on the following score ranges: ≥ 90%, 80–89.99%, 60–79.99%, 40–59.99%, and <40%, respectively [24].
- Skill: The skill questions were employed to categorize the level of skill as excellent, very good, good, insufficient, or poor based on the following score ranges: ≥ 90%, 80–89.99%, 70–79.99%, 50–69.99%, and <50%, respectively [24].

### Data collection tool and procedure

A self-administered questionnaire comprised of three sections: demographic and educational characteristics, adopted 26 knowledge, and 12 skill testing questions [24]. The first section gathered demographic and educational characteristics of participants, including age, gender, year of study, study program, and any training received related to children’s environmental health (CEH).

The second section included 26 knowledge-testing questions, with 24 items demonstrating a high reliability of 0.98 [24]. These items were validated by five experts in pediatric nursing and nursing education, achieving a reliability score of 0.89. The questions underwent content validity assessment and were modified to incorporate locally relevant terminology where necessary. The knowledge testing utilized three response options: True, False, and “I don’t know,” with the latter included to reduce guessing. Nursing students received a score of one [1] for correct answers and zero (0) for incorrect responses or if they selected “I don’t know.”

The third section focused on a skills questionnaire related to children’s environmental health, comprising 12 items that showed good fit and a reliability score of 0.87 [24]. This section is also validated by the same experts who conducted the content validity assessment for the knowledge questionnaire and demonstrated a reliability of 0.84. The skill testing responses were captured using the five-point Likert scale, ranging from “strongly agree” to “strongly disagree.” For positively stated statements, a value of five was assigned for “strongly agree,” four for “agree,” three for “neither agree nor disagree,” two for “disagree,” and one for “strongly disagree.” Conversely, for negatively stated statements, the values were reversed.

### Data quality control

A pretest was conducted at the RVU Gimbi campus, located west of the main town (Nekemte). Minor adjustments were made based on the pretest findings. For the study, five data collectors and two supervisors were recruited and received a comprehensive two-day training on the principles of data collection and handling. The principal investigator conducted nightly checks to ensure the consistency and completeness of the data. The time to complete filling out the questionnaire is estimated based on the time the experts allocated (15–30 minutes) and the minimum (15 minutes) and maximum time (25 minutes) the participants utilized in the pretest.

### Data processing and analysis

Following data collection, the information was cleaned and checked for completeness and consistency using Epi Data version 3.1. Subsequently, the data was exported to SPSS version 25 for analysis. Descriptive statistics were employed to present the findings in narrative and tabular forms. Linear regression was utilized to identify the predictors of nursing students’ knowledge and skills about CEH. In this model, a significance level of p < 0.05 with a 95% confidence interval were used to identify statistically associated factors in multivariable linear regression.

### Ethical considerations

Ethical clearance was obtained from the school of nursing ethical review committee at Wollega University, the college of medicine and health sciences, and the institutional research review board (HIS RERC/112/2023), given on 18^th^ April 2023. Then an official letter was written to each campus for permission and support; however, participants have the right to accept or deny participating in the study. Written and verbal informed consent was obtained prior to data collection. Participant confidentiality was ensured by maintaining the anonymity of the questionnaires in a self-administered format. Students participating in the study were free to withdraw from completing the questionnaires if they felt uncomfortable. The questionnaires were filled out in their respective classrooms.

Students were informed that completing the study would take approximately 15–30 minutes. All methods adhered to relevant guidelines and regulations. No identifying information was collected from the students, ensuring confidentiality throughout the process. They were instructed not to use any reading materials or discuss the questions with peers while answering. Upon completion, students were asked to place their questionnaires in a designated envelope, which was sealed immediately.

## Results

### Demographic and educational characteristics of the participants

In this study, a total of 634 students were invited to participate, of which 589 completed the questionnaire, yielding a response rate of 93%. The mean age of the participants was 24.76 years, with a standard deviation of 4.97 years (24.76 ± 4.97). The gender distribution was nearly equal, with approximately 50% male and 50% female participants. Moreover, 68% of the participants were in their third year of study, while 20.5% were in their fourth year. The majority of the participants were enrolled in comprehensive nursing programs, constituting over three-fourths of the sample. Additionally, 64% of the students reported not having received any training or lectures on children’s environmental health, while the remaining proportion had undergone such training (Table 1).

**Table 1:** Characteristics of nursing students, Nekemte town, Ethiopia, 2023.

Variables	Category	Frequency	Percentage (%)
Age (24.76 ± 4.97) (years)	20-26	440	74.8%
	>26	149	25.3%
Gender	Male	311	52.8
	Female	278	47.2
Study year	First-year	54	9.2
	Second-year	8	1.4
	Third-year	406	68.9
	Fourth-year	121	20.5
Study program	Comprehensive nursing	455	77.2
	Specialty nursing	134	22.8
Received training/lecture about CEH	Yes	373	63.3

### Knowledge of the nursing students about children’s environmental health

**Fig 1:**
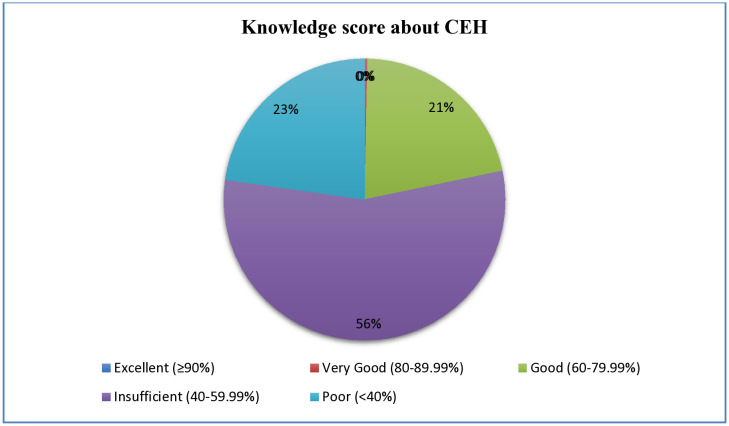
Level of Knowledge of nursing students about children’s environmental health, Nekemte town, Ethopia, 2023.

The study revealed that the majority of nursing students demonstrated insufficient knowledge about children’s environmental health (56%), with an additional 23% exhibiting a poor level of knowledge, and none scoring ≥90% (Fig 1).

Furthermore, a significant number of students provided correct responses to items assessing risk factors for respiratory diseases (81.7%) and asthma (81.7%). Conversely, fewer students demonstrated knowledge regarding the effects of nitrogen oxide and tobacco smoke on the skin (22.8%), and identified schools and nurseries (16.5%) and parks and gardens (12.2%) as less hazardous environments (Table 2).

**Table 2:** Nursing students’ knowledge about CEH, Nekemte town, Ethiopia, 2023.

Items	Correctly answered (%)
1. The pediatric population is more susceptible to environmental threats due to their biological immaturity*.	167 (28.4)
2. The increased energy and metabolic consumption of the pediatric population protects children from environmental hazards.	167 (28.4)
3. The higher rate of cell growth during the pediatric age increases the risk of health effects caused by environmental factors*.	256(43.5)
4. Environmental factors do not influence hormonal secretion during puberty.	394(66.9)
5. Nitrogen oxide from fossil fuels in the home and tobacco smoke causes redness and burns on the skin.	134(22.8)
6. Particles from animals exacerbate asthma crisis^*^.	481(81.7)
7. Increased humidity at home improves respiratory diseases in children.	481(81.7)
8. Passive smoking is associated with the development of acute leukemia in children^*^.	345(58.6)
9. Childhood leukemia incidence rates are higher in the areas most exposed to radon^*^.	274(46.5)
10. Overexposure to solar ultraviolet radiations can damage the skin of adults more severely than that of children.	291(49.4)
11. During childhood more than half of the expected lifetime solar ultraviolet radiation is absorbed^*^.	326(55.3)
12. Lead accumulates in the body affecting the nervous system^*^.	348(59.1)
13. Chronic dietary exposure to mercury (fish and shellfish) is less toxic to children’s central nervous system than to adults.	258(43.8)
14. Exposure to pesticides increases the risk of developing attention deficit problems in school-aged children*.	356(60.4)
15. Children born to smoking mothers during pregnancy are at risk of lower intellectual capacity*.	469(79.6)
16. Exposure to organic solvents during fetal development can cause learning disabilities in children*.	351(59.6)
17. Water containing nitrates can only cause intoxication during childhood.	330(56.0)
18. Chlorination of water forms sub-products from the disinfection process that have been classified as carcinogenic*.	188(31.9)
19. The major source of childhood exposure to pesticides is through ambient air.	194(32.9)
20. The main route of exposure to mercury is through cereal intake.	295(50.1)
21. Exposure to lead through diet occurs mainly through fish intake.	287(48.7)
22. Food colorings and preservatives are associated with central nervous system problems*.	256(43.5)
23. Genetically modified foods cause fewer allergic reactions in children.	170(28.9)
24. Schools and nurseries are environmentally safe places.	97(16.5)
25. Children are exposed to higher concentrations of air pollutants at home than outdoors.	298(50.6)
26. Parks and gardens are the areas with the least environmental pollutants where children can play.	72(12.2)

Key: * represents items whose correct answer is true

### Skills towards children’s environmental health

**Fig 2:**
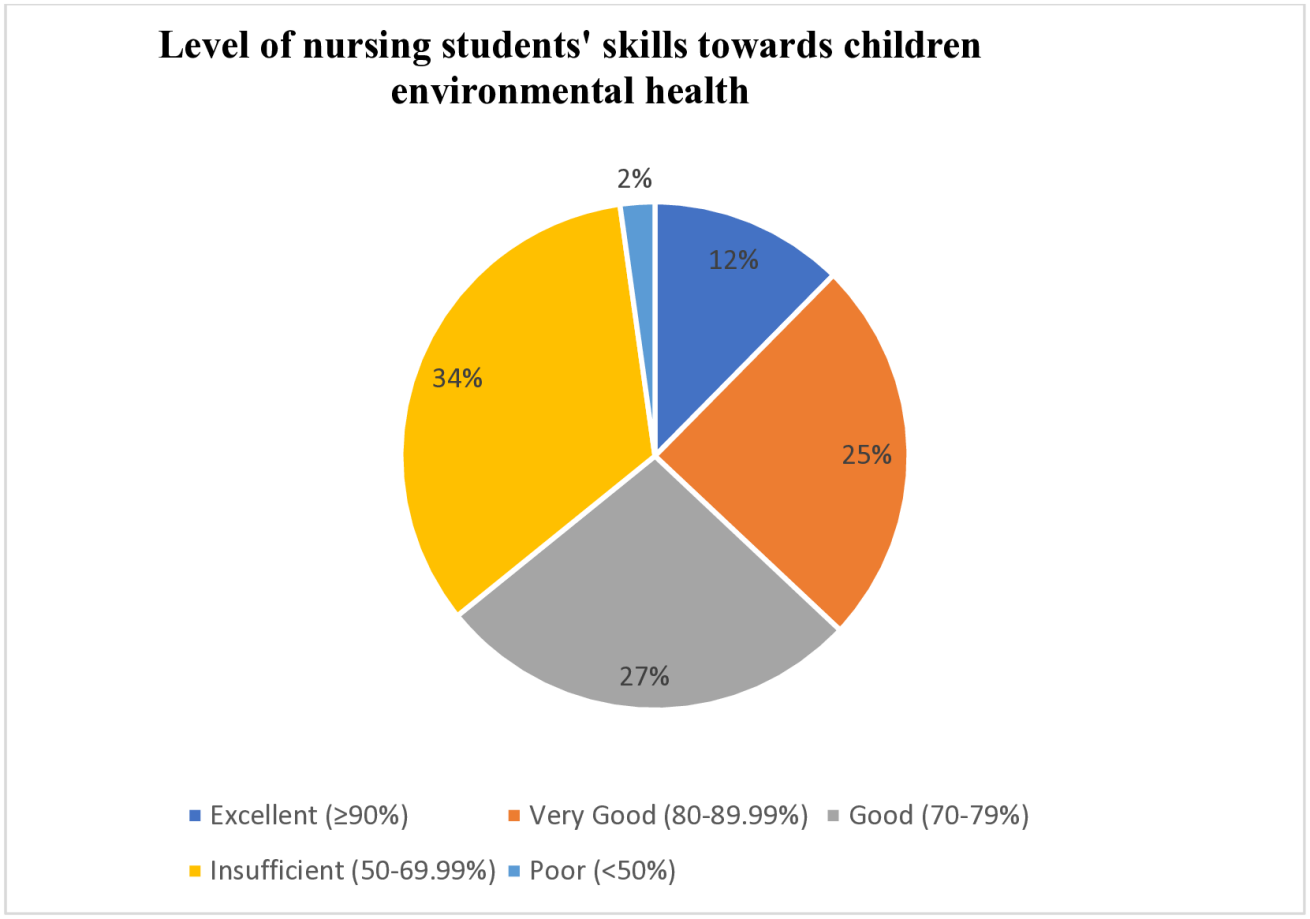
Level of nursing students’ skills performance towards children’s environmental health, Nekemte town, Ethopia, 2023.

In this survey, 34% of participants demonstrated insufficient skill in addressing children’s environmental health, while 25% and 27% of the learners exhibited good and better levels of proficiency in preventing children from ecological hazards, respectively (Fig 2).

The majority of students displayed higher scores for their skills in identifying environmental risk factors to which children are exposed (81.7%) and risk factors for diseases associated with the environment (71.2%). Similarly, more than half (58.3%) of the nursing students expressed their preparedness to work in a Pediatrics Environmental Health Specialty Unit (Table 3).

**Table 3:** Skills of nursing students towards CEH, Nekemte town, Ethiopia, 2023.

Items	Responses for items				
	SDA	DA	N	AG	SA
1. I am able to assess the main environmental risks to which a child is exposed.	45 (7.6)	37(6.3)	26(4.4)	316(53.7)	165(28.0)
2. I am NOT able to identify the environmental risks that can cause respiratory diseases in a child.	180(30.6)	239(40.6)	59(10.0)	62(10.5)	49(8.3)
3. I am able to identify the environmental risks that can cause neoplastic diseases in a child.	60(10.2)	108(18.3)	89(15.1)	238(40.4)	94(16.0)
4. I am NOT able to identify the environmental risks that can cause neurological disorders in a child.	101(17.1)	220(37.4)	85(14.4)	137(23.3)	46(7.8)
5. I am able to provide health education to parents about the main contaminants in their child’s food.	48(8.1)	36(6.1)	38(6.5)	258(43.8)	209(35.5)
6. I am NOT able to identify the environmental risks in playgrounds.	204(34.6)	223(37.9)	53(9.0)	61(10.4)	48(8.1)
7. I am able to provide health education to parents about actions to minimize environmental risks to which a child is exposed when playing outdoors.	60(10.2)	51(8.7)	35(5.9)	229(38.9)	214(36.3)
8. I am NOT able to identify the environmental risks in a child’s home.	199(33.8)	213(36.2)	54(9.2)	66(11.2)	57(9.7)
9. I am able to provide health promotion to parents about environmental risks at home.	55(9.3)	65(11.0)	40(6.8)	237(40.2)	192(32.6)
10. I am able to identify the environmental risks in a child’s school.	48(8.1)	53(9.0)	34(5.8)	244(41.4)	210(35.7)
11. I am NOT able to identify the actions needed to combat environmental risks in a child’s school.	191(32.4)	224(38.0)	57(9.7)	67(11.4)	50(8.5)
12. I do not feel able to do my job as a nurse in a Paediatrics Environmental Health Specialty Unit.	150(25.5)	193(32.8)	62(10.5)	120(20.4)	64(10.9)

### Keys: SDA: Strongly disagree; DA: Disagree; Neither agree nor disagree (neutral); AG: Agree; SA: Strongly agree

### Predictors of nursing students’ knowledge and skill in children’s environmental health

We employed the linear regression to identify the determinants of nursing students’ knowledge and skills in CEH. The analysis revealed that older age, male gender, receipt of training or lectures on CEH, and higher skill scores in CEH were associated with increased odds of achieving a high expertise score among nursing students. Conversely, being a senior student and studying comprehensive nursing were associated with increased odds of achieving a low knowledge result. Additionally, age and attitude scores of nursing students were significantly associated with their knowledge of CEH (Table 4). The odds of older students achieving high knowledge results were 1.06 times greater than those of younger students, with a 51.45% probability for older students to have a high knowledge level. Furthermore, students who demonstrated high skills in CEH had 1.02 times the odds of those who demonstrated low skills in CEH (50.66%) (Table 4).

**Table 4:** Predictors of nursing students’ knowledge and skills regarding CEH in Nekemte town, Ethiopia, 2023.

Predictors of nursing students’ knowledge about CEH						
Variables	Category	B	P-value	Exp(B)	CI 95%	
					Lower	Upper
Gender	Male	0.182	0.269	1.199	0.869	1.656
	Female	0^a^	.	1	.	
Year of study	First-year	−0.305	0.412	0.737	0.355	1.529
	Second-year	0.078	0.913	1.081	0.267	4.382
	Third-year	−0.405	0.050	0.667	0.445	1.001
	Fourth year	0^a^	.	1	.	
Received training/lecture about CEH	Yes	0.112	0.604	1.119	0.732	1.710
	No	0^a^	.	1	.	
Nursing study type	Comprehensive	−0.275	0.244	0.759	0.478	1.206
	Specialty	0^a^	.	1	.	
Age	Age	0.060	0.001*	1.061	1.024	1.100
Skills of students	Skills score	0.026	0.016*	1.027	1.005	1.048
**Predictors of nursing students’ skills about CEH**						
Gender	Male	0.513	0 .001	1.671	1.236	2.257
	Female	0^a^		1	.	
Year of study	First-year	0.496	0.152	1.642	0.833	3.238
	Second-year	−0.927	0.178	0.396	0.103	1.525
	Third-year	0.078	0.686	1.081	0.741	1.576
	Fourth year	0^a^	.	1	.	
Received training/ lecture about CEH	Yes	−0.105	0.608	0.900	0.601	1.347
	No	0^a^	.	1	.	
Nursing study type	Comprehensive	0.117	0.595	1.124	0.730	1.731
	Specialty	0^a^		1		
Age	Age	0.054	0.002*	1.056	1.020	1.092
Knowledge of students	knowledge score	0.059	0.016*	1.060	1.011	1.112

Keys: 0^a^: Reference variable, *: significant variable at p-value less than 0.05

Similarly, the odds of a higher score towards CEH skills are reported among students who are aged and male, attending their first and second year classes, studying a comprehensive course, and scoring a better result about CEH knowledge. The low scores in skills were found among the students who received training or lectures about CEH. However, a statistically significant difference is shown between the levels of skills and age, gender, and knowledge score.

In terms of nursing students’ skills, the odds of male nursing students achieving a favorable level of skill are 1.67 times higher than the same odds for female nursing students, with a 62.54% probability for male nursing students to score favorably in CEH skills. Additionally, the odds of older students achieving high skill results are 1.05 times greater than the odds for younger students, with a 51.36% probability for older students to have a high skill level. Moreover, students with a higher level of knowledge about CEH have 1.06 times higher odds of scoring better than students with a lower level of knowledge, with a 51.45% probability for students who scored high in skills (Table 4).

## Discussion

The present study aimed to evaluate nursing students’ knowledge and skills regarding children’s environmental health (CEH) in Nekemte town, Ethiopia. The results indicated that a significant majority, 79%, of the nursing students exhibited poor (23%) or insufficient (56%) knowledge of CEH, while only 21% demonstrated good knowledge. In terms of skills, 27% of the students were classified as having good skills, 25% as very good, and 12% as excellent in their CEH-related skills. Additionally, the study identified students’ age and skills as predictors of their knowledge scores, while both age and knowledge significantly influenced their skill levels.

The susceptibility of children to environmental hazards demands a comprehensive education for nursing students on the subject of children’s environmental health, as it enables them to effectively prevent and mitigate the detrimental effects [25–27]. Nurses possess a distinct advantage in identifying environmental hazards that impact children [18,28,29]. The current study reveals that nursing students’ level of knowledge is lower than the study findings in the USA (79.4%) and England (77.59%) of nursing student knowledge regarding children’s environmental health (CEH) [30,31]. Given that environmental and climate change concerns are more prominent in developed countries compared to developing nations, it is likely that the awareness and curriculum content for nursing students in these regions are more robust. The difference might be due to the large sample size undertaken by the counterparts, variations in the cutoff points and their levels of category, and variation in the study tools. Similarly, the findings of this study were contradictory with the study conducted in Croatia (58.49%) [32], which focused on assessing the nursing students’ knowledge.

Furthermore, approximately one-third (34%) of the study participants had insufficient skills in relation to children’s environmental health. This finding aligns with a study conducted in England, which reported similar results (33.62%) [31]. However, the finding was lower than the study conducted in USA (47.2%) [30]. The study demonstrated that a favorable neonatal intensive care environment, combined with clustered nursing attention, significantly enhanced the vital indicators of neonates. This improvement was evident in several key metrics, including respiration rates, heart rates, oxygen saturation levels, and systolic blood pressure, indicating a positive impact on the overall health and stability of the infants in care. Likewise, it enhanced sleep time while decreasing wakefulness and pain score [33]. Demonstrating the nursing skill during the nursing study period is crucial for understanding the impact of the care environment on the children’s health and for practicing skills in future nursing settings.

The study revealed that the age and attitude scores of nursing students significantly predicted their knowledge of children’s environmental health. This is in harmony with the study findings in Spain [30]. Additionally, the findings indicated a strong alignment between the students’ knowledge and their attitude scores, suggesting that those with a positive attitude were better able to process and apply the information they had learned, demonstrating a clearer understanding of the relevant facts, principles, and rules [34]. The positive correlation between age and CEH knowledge may be attributed to the accumulation of life and clinical experiences, which enhance critical thinking and the application of theoretical knowledge in practical settings. Older students often possess greater maturity and a more comprehensive understanding of health issues, enabling them to recognize and address environmental risks more effectively. Additionally, future studies should incorporate healthcare nurses to explore further significant factors contributing to the observed knowledge gaps. It guides the importance of inclusive learning environment for all nursing students.

In the present study, the method analysis identified significant associations between the skill of nurses in relation to children’s environmental health and certain demographic and educational factors. Nursing students’ knowledge score is increased with their skills in CEH. The study done in Spain also displayed similar findings [30]. It has been observed that male nursing students may employ more hands-on, problem-solving approaches to learning, facilitating better retention and application of knowledge in skill-based domains [35]. Therefore, by emphasizing practical problem-solving, these learning methods may improve assessment outcomes, especially in domains like children’s environmental health that demand applied skills and critical thinking.

Furthermore, this study also indicated that male nursing students tend to have higher knowledge scores than female nursing students. Male nursing students may achieve higher knowledge scores due to a combination of practical learning strategies, clinical exposure, confidence, and motivational influences, all of which enhance understanding and retention of theoretical and applied concepts [35]. Male students may benefit from more concentrated clinical experiences during training, which helps them link theoretical knowledge to practical application. Social and cultural expectations to demonstrate competence may further motivate male students. Motivation and positive learning attitudes are recognized as strong predictors of success in healthcare education [36]. To address these disparities, further research is needed into educational practices, societal influences, and personal experiences. Such insights are critical for fostering an inclusive learning environment that supports all nursing students regardless of gender in excelling across all aspects of pediatric health, including environmental health considerations.

The findings indicate that male nursing students are more likely to achieve favorable skill scores compared to their female counterparts. The observed gender differences in environmental health skills among nursing students are multifaceted, stemming from educational experiences, emotional factors, and societal influences. Another studies have reported that male students scored higher on environmental health skills assessments [30,37,38], suggesting that gender differences in educational experiences and curriculum design may influence these outcomes [37]. It has also shown that male nursing students often report lower levels of emotional distress compared to female students [39]. Lower anxiety levels may facilitate better focus and performance in skill-based assessments, potentially contributing to the observed gender differences in environmental health skills.

Additionally, older nursing students, aged >26 years, demonstrate their skills in protecting children from environmental hazards. The older nursing students may have more life experience or previous exposure to health care settings. In this study, many nursing students are attending their Bachelor of Science degree after they worked in the diploma or level three and above in nursing profession. The study also suggested that hands-on experience enhances the ability to apply theoretical knowledge effectively [40]. Furthermore, Students who are more knowledgeable in CEH demonstrate slightly but significantly better practical skills. A study conducted among 308 nursing students in Spain found that greater knowledge of CEH was associated with improved skills in protecting children from environmental risk [24]. This indicates knowledgeable students can recognize risks early, make informed decisions, and integrate environmental health with broader child care practices, enhancing their practical skills. This integration enhances their ability to apply theoretical knowledge effectively in real-world scenarios.

A cross-sectional study design captures data at a single point in time, which limits the ability to establish causal relationships between outcomes and explanatory variables. This study included nursing students with varying levels of experience (those upgrading their education often have extensive healthcare experience, while generic nursing students have differing levels of clinical exposure, ranging from single to multiple practical sessions) which could influence their scores. Another potential limitation is information diffusion bias, even though students were informed about the importance of not sharing information. Furthermore, as the study focused exclusively on nursing students in Nekemte town, the generalizability of the findings to all nursing students in Ethiopia may be limited. Students’ knowledge and attitudes may also be affected by factors such as hospital environments, academic experiences in lectures and practical sessions, and other contextual variables. Finally, the use of self-reported data introduces subjectivity and the possibility of both over-reporting and under-reporting responses.

## Conclusions

The study revealed that nursing students possess unsatisfactory knowledge and skills regarding children’s environmental health. Factors such as the students’ age for both knowledge and skills, knowledge and skills for each other were significantly associated with their understanding of this topic. It is essential to cultivate an inclusive learning environment that supports all nursing students. Given the limitations of cross-sectional study designs, which do not effectively establish causal relationships, it is advisable to conduct research involving nursing students from various geographical regions in Ethiopia. Additionally, future studies should incorporate healthcare nurses to explore further significant factors contributing to the observed knowledge gaps.

To address this gap, it is strongly recommended to integrate children’s environmental health into the nursing curriculum (pediatrics and neonatal nursing modules) and to encourage nursing students to enhance their understanding and improve their skills in this vital area. Pediatric and neonatal nurse educators play a crucial role in integrating their expertise into the curriculum, collaborating effectively to enhance students’ understanding and development. Further research on a national level or across different regions within Ethiopia to explore broader trend is suggested.

## Abbreviations

CEH: Children’s Environmental Health
KM: Kilometer
ID: Identification
NGUC: New-Generation University College
RVUC: Rift Valley University College
WU: Wollega University.
